# A Preservative against Syphilis

**Published:** 1847-10

**Authors:** 


*A Preservative against Syphilis.*—It is announced in a late number of *L'Union Médicale*, that a M. Debrosse professes to have discovered a method of preservation against attacks of syphilis. The inventor states "that any part plunged for five minutes in the prophylactic liquid may be exposed with impunity to the contact of mucous membrane impregnated with the virus of syphilis." A mercantile house has made a proposition to the Government of Spain on the subject; and it has been so far seriously entertained that the Government has referred the question to the decision of the Academy of Medicine and Surgery of New Castle. While the discussion was proceeding, (with closed doors,) a Dr. Lafond, of Bayonne, accompanied by a member of the commercial house in question, appeared before the Academy, and read a memoir on the success which had attended the use of Debrosse's liquid, stating that it was an astringent preparation. A committee was therefore appointed to inquire into the matter, and according to the latest intelligence, the members of this committee were busily engaged in carrying out their investigations on this curious subject.—*Ibid, from L'Union Médicale.*

